# Cancer-related psychosocial factors and self-reported changes in lifestyle among gynecological cancer survivors: cross-sectional analysis of PROFILES registry data

**DOI:** 10.1007/s00520-021-06433-0

**Published:** 2021-08-28

**Authors:** Karin A. J. Driessen, Belle H. de Rooij, M. Caroline Vos, Dorry Boll, Johanna M. A. Pijnenborg, Meeke Hoedjes, Sandra Beijer, Nicole P. M. Ezendam

**Affiliations:** 1 The Netherlands Comprehensive Cancer Organisation, Utrecht, The Netherlands; 2 Department of Medical and Clinical Psychology, Tilburg University, Tilburg, The Netherlands; 3 Department of Obstetrics and Gynecology, Elisabeth-TweeSteden Hospital, Tilburg, The Netherlands; 4 Department of Gynecology, Catharina Hospital, Eindhoven, The Netherlands; 5 Department of Obstetrics & Gynecology, Radboud University Medical Center, Nijmegen, The Netherlands

**Keywords:** Cancer survivors, Gynecology, Health behavior, Diet, Exercise, Psychosocial factors

## Abstract

**Purpose:**

Obesity is prevalent in gynecological cancer survivors and is associated with impaired health outcomes. Concerns due to cancer and its treatment may impact changes in lifestyle after cancer. This study aimed to assess the association between cancer-related psychosocial factors and changes in physical activity and diet, 18 months after initial treatment among gynecological cancer survivors.

**Methods:**

Cross-sectional data from the ROGY Care study were used, including endometrial and ovarian cancer patients treated with curative intent. The Impact of Cancer Scale (IOCv2) was used to assess cancer-related psychosocial factors. Self-reported changes in nutrients/food groups and in physical activity post-diagnosis were classified into change groups (less/equal/more). Multivariable logistic regression models were used to assess associations.

**Results:**

Data from 229 cancer survivors (59% endometrial, 41% ovarian, mean age 66 ± 9.5, 70% tumor stage I) were analyzed. In total, 20% reported to eat healthier from diagnosis up to 18 months after initial treatment, 17% reported less physical activity and 20% more physical activity. Health awareness (OR 2.79, 95% CI: 1.38; 5.65), body change concerns (OR 3.04 95% CI: 1.71; 5.39), life interferences (OR 4.88 95% 2.29; 10.38) and worry (OR 2.62, 95% CI: 1.42; 4.85) were significantly associated with less physical activity up to 18 months after initial treatment whereby gastrointestinal symptoms were an important confounder.

**Conclusion(s):**

This study underlines the need to raise awareness of the benefits of a healthy lifestyle and to provide tailored lifestyle advice, taking into account survivors’ health awareness, body change concerns, life interferences, worry and gastrointestinal symptoms, in order to improve health behavior among gynecological cancer survivors.

**Trial Registration:**

http://clinicaltrials.gov Identifier: NCT01185626, August 20, 2010

**Supplementary Information:**

The online version contains supplementary material available at 10.1007/s00520-021-06433-0.

## Introduction

The development of endometrial cancer and (to a smaller extent) ovarian cancer can be attributed to consequences of obesity in many cases. Consequently, obesity prevalence rates are high in survivors of endometrial (45–70%) and ovarian (30%) cancer [1, 2]. Moreover, removal of the ovaries is part of standard surgical treatment in gynecological cancer and has been associated with increased visceral fat accumulation and body fatness [3]. Obesity has been associated with impaired quality of life developing health problems (e.g. cardiovascular disease) and worse overall survival [2, 4–6]. To reduce obesity, it is important to promote physical activity and healthy dietary behavior [7].

A cancer diagnosis has been suggested to be a ‘teachable-moment’ which has the potential to motivate patients to make healthy lifestyle changes [8]. Even though 30–60% of cancer survivors change their lifestyle post-diagnosis, adherence to recommendations for physical activity and diet remains relatively low as compared with the cancer-free population [9, 10]. Moreover, adherence to recommendations among cancer survivors has been found to decrease over time [11]. These findings suggest that efforts are needed to encourage healthy behavior among cancer survivors.

According to theories and models of health behavior change, such as the Health Belief Model (HBM), perceived barriers, such as fatigue, decreased physical functioning and body image concerns, have shown to be important aspects in explaining behavior (change) [12, 13]. In addition, fear and worry for cancer recurrence have been related to intentions to change health [14]. In line with the HBM, people who perceive susceptibility to a particular health problem have been shown to be more likely to engage in risk-reducing health behavior [13]. On the other hand, distress has been related to unhealthy behavior, possibly due to negative coping strategies [14]. Furthermore, increased sense of meaning or purpose may increase health awareness, which is a prerequisite for behavior change, while posttraumatic growth may be a cue to action, as described in the HBM [13, 15–17].

Current studies explaining health behavior change after cancer diagnosis focus on more general aspects including socio-demographic factors, clinical variables or cognitive behavioral factors [9, 14, 18, 19]. These studies neither focus on change of health behavior comparing pre-diagnosis nor distinguish associations of factors with physical activity and diet [9, 14, 18, 19]. Insight into associations between cancer-related psychosocial factors and lifestyle changes may help identifying factors that may explain engagement in health behavior change. This will contribute to development of more targeted lifestyle interventions to promote health behavior change among cancer survivors. Therefore, this study aims to examine the association between cancer-related psychosocial factors and changes in lifestyle after diagnosis (diet) or treatment (physical activity) compared to 18 months after initial treatment among gynecological cancer survivors.

## Methods

### Study design

This study is a secondary cross-sectional analysis of data from the ROGY Care trial [20]. The ROGY Care trial is a pragmatic, cluster randomized controlled trial that aimed to assess the effect of a Survivorship Care Plan (SCP) on information provision and post-treatment care for cancer patients [20]. Data from both trial arms were analyzed together, as it is assumed that the SCP did not affect lifestyle because it did provide minimal information on lifestyle. The questionnaire administered at 18 months after initial treatment was used for the purpose of this study. The study was approved by the Medical Research Ethics Committee [20].

### Study population and data collection

Eligibility criteria to participate in the ROGY Care trial were (1) newly diagnosed patients with either primary endometrial or ovarian cancer between April 2011 and October 2012, (2) curative treatment, (3) aged ≥ 18 years and (4) being able to complete a Dutch questionnaire. In total, 544 participants (*N* = 296 endometrial, *N* = 248 ovarian) were eligible, and 73% responded (*N* = 221 endometrial, *N* = 174 ovarian) to the baseline questionnaire (Online Resource 1). Patients were included immediately after initial surgery. A total of 230 participants (*N* = 137 endometrial, *N* = 93 ovarian) completed the questionnaire at 18 months that was used for the analyses in this paper (response of 42% from eligible participants for the study) [20]. At that time, all patients had completed primary treatment.

## Measures

### Cancer-related psychosocial factors

The validated Impact of Cancer Scale version 2 (IOCv2) was used to assess cancer-related psychosocial factors [21]. For each of the subscales: health awareness, meaning of cancer, appearance concerns, body change concerns, life interferences and worry (including items scored on a 5-point Likert scale), a sum score was calculated, whereby a higher score referred to more problems or concerns. The item ‘having had cancer has made me take better care of myself’ from the subscale health awareness was excluded from the analysis as Cronbach’s alpha improved when excluding this item (from 0.79 up to 0.84). Furthermore, it was strongly related to our dependent variables (change in lifestyle). Internal consistency (Cronbach’s alpha) for each of the subscales was good (ranging from/range 0.83–0.93).

### Changes in dietary behavior and physical activity

By use of self-developed questionnaires from the PROFILES registry studies, participants reported if they made changes in diet since they were diagnosed with cancer (yes/no) until 18 months after initial treatment [22]. If so, they reported their most important changes since diagnosis (more/less consumption), per type of food (fat, meat, fish, vegetables, fruit, milk and dairy products, dietary fibers/whole grain products, sugar, alcohol, water, salt and ‘others’). Changes were classified as healthy (more consumption of fruit, vegetables, fibers/whole grain and less consumption of fat, meat, sugar, alcohol and salt) or unhealthy (vice versa) according to the World Cancer Research Fund (WCRF) recommendations [23]. If respondents reported more healthy changes than unhealthy changes, they were classified into the eating healthier (*N* = 44) group and if they reported more unhealthy changes, as many unhealthy as healthy changes or no change, they were classified into the no change/eating unhealthier (*N* = 172) group. As only few participants made unhealthy choices (*N* = 9), these individuals were grouped with the no change group.

Furthermore, participants reported if they made changes in their physical activity since they completed their cancer treatment (yes/no) compared to pre-diagnosis. If so, participants were able to report for several types of physical activities (walking, cycling, gardening, housekeeping, sports and others), per type, if they increased or decreased their activity level. Subsequently, respondents were classified into the group: no change in physical activity (*N* = 133), more physical activity (*N* = 43) or less physical activity (*N* = 36), according to the amount of reported decreases or increases in physical activity (despite differences in intensity of activities).

### Socio-demographic and clinical factors

Factors obtained from the Netherlands Cancer Registry were age and socio-economic status (based on postal code of the residence area of the patient, categorized into low/ intermediate vs. high) and clinical factors (cancer type: ovarian/endometrial; tumor stage: I vs. II, III or IV; type of treatment: surgery, radiotherapy and chemotherapy; amount of comorbidities: 0,1, > 1) [24]. Self-reported questionnaires included marital status (married/living together vs. divorced, widowed, never married/never lived together), educational level (low, intermediate, high) and physical limitations in daily activities (scored as 100 minus score of the EORTC QLQ-C30 physical functioning scale) [25]. Body Mass Index (BMI; weight in kg/length in m^2^) at 18 months after initial treatment was based on self-report. Reasons for change in physical activity included complaints related to cancer or its treatment (not further specified), support of physical recovery, prevention of cancer recurrence and other reasons. Furthermore, participants were asked whether they received advice (yes/no) regarding diet (and/or dietary supplements) and/or physical activity, from diagnosis up to 18 months thereafter (provider or moment of received advice was not further specified). Gastrointestinal (GI) symptoms were obtained from a questionnaire developed by a dietitian from our research team and included unintentional weight loss or gain, poor appetite, cramps, frequent small amounts of stool, constipation, diarrhea, change in taste and/or smell, aversion, acid reflux and others. Unintentional weight loss and weight gain were excluded, and a sum score (range 0–11) was based on the number of symptoms (suffered in the past or still suffering).

### Statistics

Descriptive data was reported by means and standard deviations (SDs) or medians and interquartile ranges (IQRs) (continuous variables), frequencies and percentages (dichotomous and categorical variables) and compared by use of *t* tests, ANOVA with post hoc procedures, Mann–Whitney U and Kruskal–Wallis tests (continuous variables) and Chi-square/Fisher’s Exact tests (categorical variables).

Statistical analyses were performed by SAS for Windows (version 9.4). Associations between psychosocial factors as independent variables (i.e. sum scores from the scales health awareness, meaning of cancer, appearance concerns, body change concerns, life interferences, worry (range 1–5)) and dietary changes (no change/eating unhealthier (reference)/ eating healthier) or physical activity changes (no change(reference)/less/more) as dependent variables were analyzed with logistic regression models. Before analyzing the data, assumptions were checked and not violated. *P* values < 0.05 were considered as statistically significant.

Potential confounders were age, socio-economic status, partner status, cancer type, tumor stage, treatment, number of comorbidities, BMI, gastrointestinal symptoms and physical limitations since these have been associated with psychosocial well-being as well as health behavior [9, 19, 21, 26, 27]. Variables gastrointestinal symptoms (sum score, 0–11) and physical limitations were considered as confounders prior to analysis. Other variables were considered to be a confounder when it caused a relevant change (a minimum of 10%) in the regression coefficient in the univariate model [28]. By hierarchical regression, the relevant confounders (determined per subscale) were added to the univariate model in the first step. Subsequently, the variable gastrointestinal symptoms was added to all models, and the variable physical limitations was added to the physical activity change models.

## Results

In total, 229 participants were included in the analyses as they responded on any of the outcome variables (Table 1). Participants were on average 66 years, 59% were diagnosed with endometrial cancer and 77% had one or more comorbidity. In total, 80% of participants responding to dietary behavior questions (*N* = 216) reported not having changed their diet (*N* = 169) or made unhealthier changes (*N* = 3), and 20% (*N* = 44) reported healthier changes in diet. For physical activity, 63% (*N* = 133) of participants responding on physical activity questions (*N* = 212) reported not having changed their physical activity level after treatment, 17% (*N* = 36) reported less physical activity and 20% (*N* = 43) more physical activity. From the participants that reported healthier changes in diet, 29% (*N* = 12) reported to be more physically active as well.

**Table 1:** Characteristics of the total study population and according to lifestyle change groups

		Dietary behavior change (*N* = 216)			Physical activity change (*N* = 212)			
	Total *N* = 229	No change/eating unhealthier *N* = 172	Eating healthier^a^ *N* = 44	*p* value^b^	No change in physical activity *N* = 133	Less physical activity *N* = 36	More physical activity *N* = 43	*p* value^b^
Age (years)				0.87				< 0.01
Mean (SD)	66 (9.5)	66 (9.9)	66 (8.5)		67 (9.0)	67 (9.6)	61 (9.2)	
Partner status				0.97				0.07
Partner No partner	184 (81) 42 (19)	138 (81) 32 (19)	35 (81) 8 (19)		104 (79) 28 (21)	26 (74) 9 (26)	39 (93) 3 (7)	
Educational level				0.54				0.27
Low Intermediate High	29 (13) 161 (72) 35 (16)	23 (14) 122 (71) 26 (15)	4 (10) 29 (69) 9 (21)		18 (14) 98 (74) 16 (12)	3 (9) 23 (66) 9 (26)	4 (9) 29 (69) 9 (21)	
Socio-economic status				0.45				0.83
Low Intermediate High	37 (17) 83 (39) 94 (44)	29 (18) 62 (38) 71 (44)	4 (10) 17 (42) 20 (49)		22 (18) 49 (39) 54 (43)	6 (18) 14 (42) 13 (39)	6 (14) 14 (33) 22 (52)	
Cancer type				0.16				0.02
Endometrial Ovarian	136 (59) 93 (41)	97 (56) 75 (44)	30 (68) 14 (32)		90 (68) 43 (32)	17 (47) 19 (53)	21 (49) 22 (51)	
Tumor stage				0.30				< 0.01
I II III IV	161 (70) 10 (4) 44 (19) 14 (6)	123 (72) 5 (3) 35 (20) 9 (5)	29 (66) 4 (9) 8 (18) 3 (7)		104 (78) 5 (4) 21 (16) 3 (2)	18 (50) 2 (6) 10 (28) 6 (17)	28 (65) 3 (7) 9 (21) 3 (7)	
Treatment (yes)								
Surgery Radiotherapy Chemotherapy	224 (98) 45 (20) 73 (32)	168 (98) 32 (19) 57 (33)	44 (100) 10 (23) 12 (27)	0.58 0.54 0.46	133 (100) 27 (20) 31 (23)	34 (94) 9 (25) 20 (56)	43 (100) 4 (9) 16 (36)	0.03 0.16 < 0.01
Comorbidities				0.86				0.16
0 1 > 1	52 (23) 54 (24) 119 (53)	41 (24) 39 (23) 91 (53)	9 (22) 11 (27) 21 (51)		34 (26) 28 (21) 69 (53)	4 (12) 6 (18) 24 (71)	11 (26) 13 (30) 19 (44)	
Physical limitations^c^				0.96				
Median (IQR)	11.1 (0–20)	8.3 (0–20)	8.3 (0–22.5)		6.7 (0–13.3)	20.0 (10.8–40.0)	6.7 (0–13.3)	< 0.01
BMI (kg/m^2^)				0.12				0.78
Median (IQR)	27.7 (24.2–32.8)	28.2 (24.3–32.8)	25.5 (22.3–33.9)		28.1 (24.6–32.8)	27.9 (23.7–31.0)	27.6 (23.9–34.7)	
Gastrointestinal symptoms^d^ (%)				0.03				< 0.01
Not suffered from Suffered from Still suffering from	65 (30) 74 (34) 77 (36)	55 (34) 48 (30) 57 (36)	7 (16) 21 (49) 15 (35)		44 (36) 36 (29) 43 (35)	2 (6) 15 (42) 19 (53)	17 (42) 16 (39) 8 (20)	
IOCv2 scale^e^ (Mean (SD))								
Health awareness Meaning of cancer Appearance concerns Body change concerns Life interferences Worry	3.59 (1.0) 2.61 (0.8) 2.24 (0.9) 2.89 (1.1) 2.33 (0.8) 3.05 (1.0)	3.55 (1.0) 2.58 (0.8) 2.23 (0.9) 2.83 (1.1) 2.30 (0.8) 3.00 (1.0)	3.77 (0.9) 2.60 (0.7) 2.13 (0.8) 2.92 (1.0) 2.29 (0.7) 3.11 (0.8)	0.19 0.91 0.54 0.60 0.94 0.50	3.43 (0.92) 2.50 (0.77) 2.14 (0.88) 2.63 (0.98) 2.14 (0.68) 2.81 (0.81)	4.23 (0.67) 2.89 (0.65) 2.68 (0.86) 3.91 (0.78) 2.99 (0.65) 3.75 (0.84)	3.56 (1.09) 2.69 (0.81) 2.17 (1.04) 2.67 (1.11) 2.32 (0.84) 3.11 (1.09)	< 0.01 0.02 < 0.01 < 0.01 < 0.01 < 0.01

*SD* standard deviation, *IQR* interquartile range

^a^Eating healthier according to WCRF dietary recommendations for cancer survivors: more consumption of fruit, vegetables, fibers/wholegrain, less consumption of fat, meat, sugar, alcohol, salt

^b^*p* values: comparison between no change/eating unhealthier vs. eating healthier, comparison between no change vs. less vs. more physical activity according to *t* tests, ANOVA, Chi-square, Fisher’s Exact tests and Mann–Whitney *U*/Kruskal–Wallis test

^c^Higher score refers to more physical limitations

^d^Gastrointestinal symptoms: poor appetite, cramps, frequent small amounts of stool, constipation, diarrhea, change in taste and/or smell, aversion, acid indigestion and others

^e^Higher score refers to more psychosocial impact (range 1–5)

Participants reporting less physical activity more often suffered from higher stage disease (i.e. stage III (28%) or IV (17%)) compared to participants reporting no change (16% stage III and 2% stage IV) or more physical activity (21% stage III and 7% stage IV). In addition, they suffered more from physical limitations (IQR 20.0 (10.8–40) vs. 6.7 (0–13.3) for both other groups) as well as gastro-intestinal symptoms (53%) comparing others (20% in more PA group, 35% in no change in PA group) (Table 1).

The most common reported reason for decreased physical activity levels was complaints related to cancer (treatment) (44%, *N* = 16). In the group that became more physically active, important reasons were support of physical recovery (65%, *N* = 28), prevention of cancer recurrence (19%, *N* = 8) and other reasons (19%, *N* = 8) (e.g. for weight loss, to relax, for fun) (not tabulated).

From the total population, 57% (*N* = 129) received no advice. The groups that did not change their diet or physical activity more often received no advice (62% *N* = 107 and 68% *N* = 90). From all groups, survivors who became less physically active since diagnosis received the most advice regarding diet (14%, *N* = 5), physical activity (33%, *N* = 12) and combined (31%, *N* = 11) (Online Resource 2).

A higher health awareness (OR 2.79, 95% CI: 1.38; 5.65), body change concerns (OR 3.04 95% CI: 1.71; 5.39), life interferences (OR 4.88 95% 2.29; 10.38) and worry (OR 2.62, 95% CI: 1.42; 4.85) was associated with less physical activity, compared to ‘no change’, after adjustment for confounders (Table 3). In all associations, gastrointestinal symptoms were found to be a stronger confounder (but associations remained significant) compared with physical limitations (data not shown). The psychosocial factors were not associated with changes in diet (Table 2) or more physical activity (Table 3).

**Table 2:** Associations of cancer-related psychosocial factors with dietary change from diagnosis until 18 months after initial treatment among gynecological cancer survivors (*N* = 216)

	Eating healthier			
IOCv2 scale (1–5)	Adjusted for socio-demographic/clinical confounders		Adjusted for socio-demographic/clinical confounders and GI symptoms	
	OR (95% CI)	*p* value	OR (95% CI)	*p* value
Health awareness	1.27 (0.87; 1.86)	0.21^a,b^	1.07 (0.71; 1.61)	0.75^a,b,e^
Meaning of cancer	0.94 (0.59; 1.49)	0.80^c,d^	0.86 (0.53; 1.39)	0.53^c,d,e^
Appearance concerns	1.03 (0.69; 1.54)	0.87^a,b^	0.92 (0.60; 1.41)	0.69^a,b,e^
Body change concerns	1.18 (0.85; 1.64)	0.32^b,c^	1.00 (0.69; 1.46)	1.00^b,c,e^
Life interferences	1.01 (0.62; 1.65)	0.97^b,d^	*	*
Worry	1.19 (0.80; 1.75)	0.39^a,b^	0.96 (0.63; 1.48)	0.86^a,b,e^

^*^Not adjusted for gastrointestinal symptoms as the content of the scale includes complaints as a consequence of cancer(treatment)

Confounders:

^a^Socio-economic status

^b^Type of cancer

^c^BMI

^d^Tumor stage

^e^Gastrointestinal symptoms (GI)

Confounders’ selection was based on relevant change in regression coefficient (> 10%) (2 most relevant confounders were added to the model) and a priori selection (gastrointestinal symptoms)

**Table 3:** Associations of cancer-related psychosocial factors with physical activity change from diagnosis until 18 months after initial treatment among gynecological cancer survivors (*N* = 212)

	Less physical activity				More physical activity			
IOC v2 Scale (1–5)	Adjusted for socio-demographic/clinical confounders		Adjusted for socio-demographic/clinical confounders, GI symptoms and/or physical limitations		Adjusted for socio-demographic/clinical confounders		Adjusted for socio-demographic/clinical confounders, GI symptoms and/or physical limitations	
	OR (95% CI)	*p* value	OR (95% CI)	*p* value	OR (95% CI)	*p* value	OR (95% CI)	*p* value
Health awareness	3.71 (2.01; 6.86)	< 0.01^a^	2.79 (1.38; 5.65)	< 0.01^a,f,g^	1.00 (0.67; 1.49)	0.68^e^	0.98 (0.61; 1.57)	0.92^e,f,g^
Meaning of cancer	1.85 (1.07; 3.18)	0.03^b^	1.43 (0.75; 2.73)	0.28^b,f,g^	1.09 (0.86; 1.38)	0.49^e^	1.59 (0.91; 2.77)	0.10^e,f,g^
Appearance concerns	1.65 (1.10; 2.47)	0.01^c^	1.31 (0.81; 2.13)	0.27^c,f,g^	0.94 (0.62; 1.41)	0.75^c^	0.78 (0.48; 1.26)	0.32^c,f,g^
Body change concerns	*	*	3.04 (1.71; 5.39)	< 0.01^f,g^	0.98 (0.68; 1.39)	0.90^b^	0.86 (0.54; 1.37)	0.53^b,f,g^
Life interferences	3.98 (2.17; 7.32)	< 0.01^d^	4.88 (2.29; 10.38)	< 0.01^d,g^	1.24 (0.75; 2.07)	0.40^b,c^	1.42 (0.77; 2.61)	0.26^b,c,g^
Worry	2.92 (1.74; 4.89)	< 0.01^d^	2.62 (1.42; 4.85)	< 0.01^d,f,g^	1.36 (0.91; 2.02)	0.13^c^	1.24 (0.77; 1.99)	0.38^c,f,g^

^*^No socio-demographic or clinical factors were identified as relevant confounders

Confounders

^a^BMI

^b^Tumor stage

^c^Type of cancer

^d^Chemotherapy

^e^Age

^f^Gastrointestinal symptoms

^g^Physical limitations

Confounders’ selection was based on relevant change in regression coefficient (> 10%) (2 most relevant confounders were added to the model) and a priori selection (gastrointestinal symptoms, physical limitations)

## Discussion

The results of this cross-sectional study indicate that, based on self-reported data, the majority of gynecological cancer survivors does not change their lifestyle after diagnosis. In contrast to favorable changes in diet or physical activity, reported decreases in physical activity were associated with cancer-related complaints. Participants who report a higher health awareness, more body change concerns, more life interferences and more worry seem to be at risk to become less physically active compared with pre-treatment up to 18 months after initial treatment. These findings are not in line with health behavior change theories, suggesting that being aware of health problems and perceiving susceptibility for health problems motivates people to engage in positive health behavior [16]. This might be explained by the use of maladaptive coping strategies (i.e. avoidance or disengagement), as these have shown to be commonly used by cancer survivors [29]. Cancer survivors focusing on their limitations and losses (i.e. higher health awareness, body change concerns and life interferences) may use a passive coping strategy, resulting in less physical activity. In addition, it has been suggested that psychological distress (including worries) is also associated with maladaptive coping responses and engaging in unfavorable health behavior [14, 30].

Furthermore, we found a relatively small proportion of cancer survivors making healthy changes in diet (20%) and/or physical activity (20%), in contrast to other studies [9, 31]. This might be explained by the fact that majority (57%) of participants received no advice regarding diet and physical activity. As there is no information on the provider and content of the advice given in the current study, it is unclear whether beneficial effects of a healthy lifestyle related to the risk of developing health problems were discussed. However, it is likely that participants receiving advice are more aware of these beneficial effects compared to participants receiving no advice and therefore were more likely to make positive health behavior changes [32]. This seems in contrast to our finding that participants reporting less physical activity more often received advice regarding physical activity, compared to other change groups. However, participants who received advice also had more physical limitations, gastrointestinal symptoms and higher impact of cancer scores compared to survivors who did not receive advice (data not shown) which might explain this unexpected finding, assuming that only participants received advice who are ‘at risk’ to become less physically active Otherwise, it is likely that participants that already have a healthy lifestyle before being diagnosed with cancer do not receive advice on lifestyle. Unfortunately, we do not have information on their lifestyle pre-diagnosis.

Furthermore, finding no associations between psychosocial impact and favorable health behavior changes might be explained by the fact that perceived benefits of physical activity were associated with increased physical activity levels instead of perceived susceptibility to health problems [33]. If survivors did not believe (or were not aware) that health behavior is important in reducing the risk of health problems, they might not change their health behavior, even if they are worried about their health or future [34].

Furthermore, associations were partly determined by the presence of gastrointestinal symptoms, as they were an important confounder. These symptoms are known side-effects of cancer treatment that sometimes rarely improve by time or even become chronic symptoms [35]. Taking into account that suffering from physical complaints (in general) was a frequently reported reason to be less physically active and physical limitations were more common in the group reporting less physical activity, attention for the potential role of (physical) complaints as a barrier in health behavior changes after cancer (treatment) is recommended. Lifestyle changes can contribute to treating complaints (e.g. physical activity in case of cancer-related fatigue) or prevent unnecessary deterioration of complaints (e.g. diet in case of bowel dysfunction) [27]. Thus, besides the importance of lifestyle advice as a motivator for engagement in healthy behavior, it has also an important role in coping with complaints and prevention of deterioration of their current lifestyle.

To the best of our knowledge, our study was the first to focus on the psychological impact of cancer in associations with both physical activity and diet in gynecological cancer survivors. Furthermore, participants were recruited by population-based sampling, and a validated scale was used to examine the psychosocial impact of cancer [21].

An important limitation of this study is that we did not have information on the magnitude and specific content of the changes in diet (e.g. amount and macronutrients) and physical activity (e.g. intensity and duration). As participants were asked to report their *most important changes* in diet, changes were assumed to be from noteworthy size.

In addition, data on diet and physical activity was self-reported by respondents which could have led to socially desirable answers and recall errors. The absence of reported unhealthy changes in diet may reflect this bias. Furthermore, due to the small sample size, models could be adjusted for a maximum of two confounders to be able to analyze models with sufficient power. In addition, participants differed by age, educational level and tumor stage compared to lost to follow-up, possibly affecting some response bias [30]. Finally, we were not able to correct for physical complaints (except gastrointestinal symptoms) due to cancer (treatment) or for received lifestyle advice. As the majority of the participants mentioned that complaints related to cancer or its treatment were a reason to change physical activity levels, this is a potential confounder.

Given the benefits of a healthy lifestyle in prevention of (other) health problems, survivors should be encouraged to live healthier. Without receiving advice, a gynecological cancer diagnosis is less likely to be utilized as a ‘teachable moment’ by cancer survivors, with regard to making healthy changes in lifestyle. For tailored lifestyle advice, oncologists (or oncology nurses) should identify survivors’ concerns and worries about their health and physical complaints (e.g. gastrointestinal symptoms) that may be barriers for engagement in health behavior and refer to other healthcare professionals if necessary. For further research, it is highly recommended to use a longitudinal study design, a larger sample and objective measurement of diet and physical activity (e.g. using nutritional diaries and accelerometers). Finally, a qualitative approach can be used to gain insight into survivors’ beliefs about lifestyle related to developing health problems, their coping strategies with cancer-related worries/concerns and their barriers for engagement in health behavior.

In conclusion, the results suggest that gynecological cancer survivors hardly improve their lifestyle after diagnosis. Some even become less physically active which was associated with complaints, which requires attention and support of oncologists. Being more aware of health, having more concerns about body changes, experiencing more life interferences and worrying more about their future or health were found to be associated with a self-reported decrease in physical activity from diagnosis up to 18 months. This study underlines the need to emphasize the benefits of a healthy lifestyle by oncologists. Moreover, oncologists should provide tailored lifestyle advice taking into account survivors’ health awareness, body change concerns, life interferences, worry and gastrointestinal symptoms, in order to improve health behavior.

## Supplementary Information

Below is the link to the electronic supplementary material.

Supplementary file1 (PDF 406 KB)

Supplementary file2 (PDF 95 KB)

Supplementary file3 (PDF 90 KB)

## Data Availability

Data is available for non-commercial use upon request (www.profilesregistry.nl).

## Code availability

Statistics code is available for non-commercial use upon request.
