# Supplementary material for: Cancer-related psychosocial factors and self-reported changes in lifestyle among gynecological cancer survivors: cross-sectional analysis of PROFILES registry data
Source: Support Care Cancer. 2021 Aug 28;30(2):1199–207. doi: 10.1007/s00520-021-06433-0 (PMC8727401; doi:10.1007/s00520-021-06433-0)
Supplement: Supplementary file 1 — Supplementary file1 (PDF 406 KB) [file 520_2021_6433_MOESM1_ESM.pdf]

Online Resource 1 Cancer-related psychosocial factors and self-reported changes in lifestyle among gynecological cancer survivors: cross sectional analysis of PROFILES registry data. *Supportive Care in Cancer*. Karin A. J. Driessen, MSc., Belle H. de Rooij, PhD, M. Caroline Vos, MD, PhD, Dorry Boll, MD, PhD, Johanna M.A. Pijnenborg, MD, PhD, Meeke Hoedjes, PhD, Sandra Beijer, PhD, Nicole P.M. Ezendam, PhD. Corresponding author: Nicole P.M. Ezendam, The Netherlands Comprehensive Cancer Organisation, n.ezendam@iknl.nl.

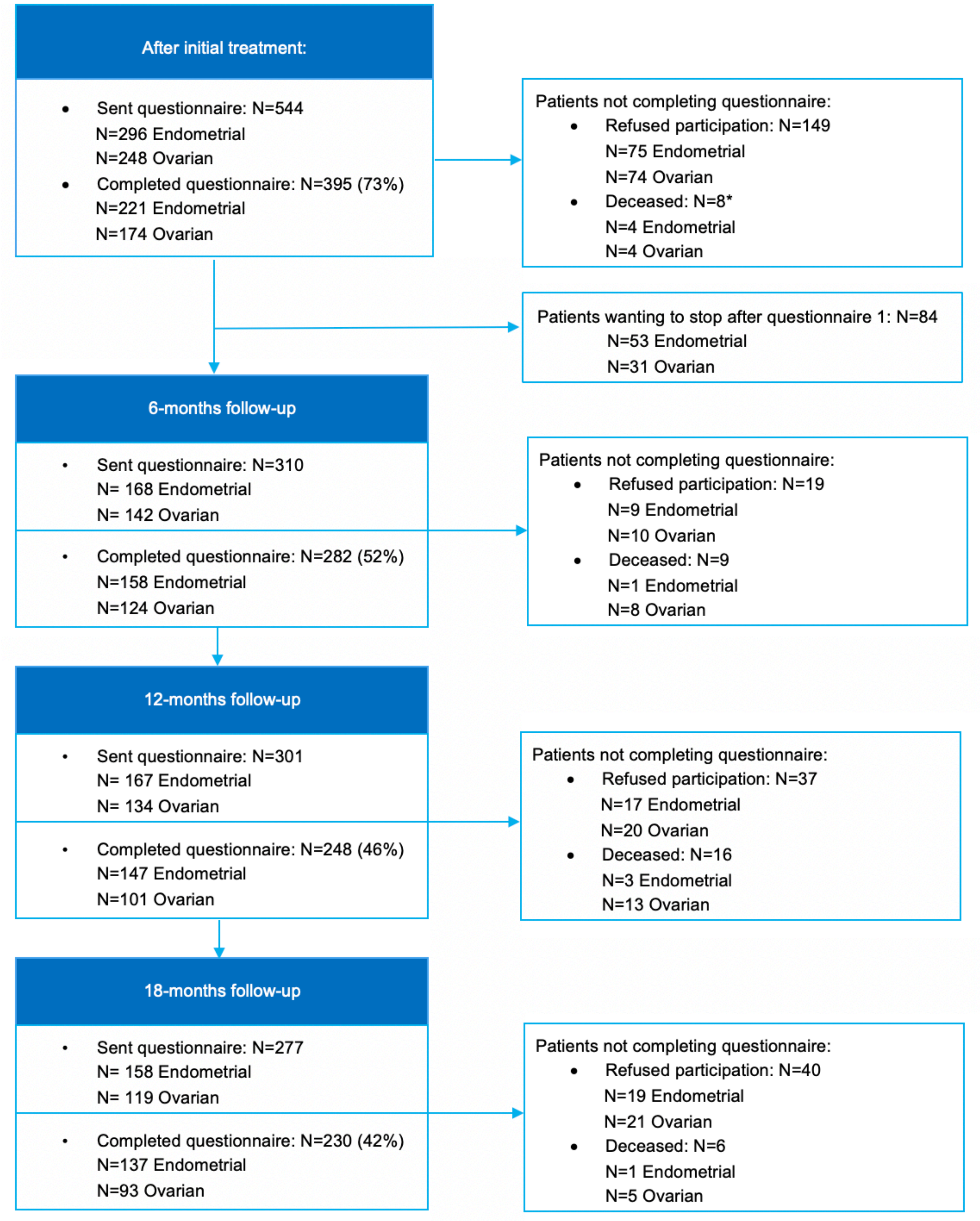

Fig. 1 Flow-chart of enrollment of participants in the ROGY Care Trial

Note: *No questionnaires were sent to those who were deceased after initial treatment or to those wanting to stop after 1 questionnaire. Patients that refused participation in a certain follow-up moment still received a questionnaire the next follow-up moment. Percentages represent the response rate with respect to the invitation after initial treatment.
