# Supplementary material for: Cancer-related psychosocial factors and self-reported changes in lifestyle among gynecological cancer survivors: cross-sectional analysis of PROFILES registry data
Source: Support Care Cancer. 2021 Aug 28;30(2):1199–207. doi: 10.1007/s00520-021-06433-0 (PMC8727401; doi:10.1007/s00520-021-06433-0)
Supplement: Supplementary file 2 — Supplementary file2 (PDF 95 KB) [file 520_2021_6433_MOESM2_ESM.pdf]

Online Resource 2 Cancer-related psychosocial factors and self-reported changes in lifestyle among gynecological cancer survivors: cross sectional analysis of PROFILES registry data. *Supportive Care in Cancer*. Karin A. J. Driessen, MSc., Belle H. de Rooij, PhD, M. Caroline Vos, MD, PhD, Dorry Boll, MD, PhD, Johanna M.A. Pijnenborg, MD, PhD, Meeke Hoedjes, PhD, Sandra Beijer, PhD, Nicole P.M. Ezendam, PhD. Corresponding author: Nicole P.M. Ezendam, The Netherlands Comprehensive Cancer Organisation, n.ezendam@iknl.nl.

Table 1. Impact of Cancer version 2 scales with item content [46].

**Scale***	**Item content**
**Positive impact domain**	
Health awareness	Having had cancer has made me more concerned about my health.<br>I do not take my body for granted since I had cancer<br>I am more aware of physical problems or changes<br>Having had cancer has made me take better care of myself
Meaning of cancer	Because of cancer I have more confidence in myself<br>Having had cancer has given me direction in life<br>Because of cancer I have become better about expressing what I want<br>Because of having had cancer I feel that I have more control of my life<br>Having had cancer turned into a reason to make changes in my life
**Negative impact domain**	
Appearance concerns	I feel disfigured<br>I sometimes wear clothing to cover parts of my body<br>I worry about how my body looks
Body change concerns	I am bothered that my body cannot do what I could before<br>I am concerned that my energy has not returned<br>Having had cancer has made me feel old
Life interferences	Uncertainty about my future affects my decisions to make plans<br>Having had cancer has made me feel alone<br>Having had cancer keeps me from doing activities I enjoy<br>I feel like cancer runs my life<br>Having had cancer has made me feel that some people do not understand me<br>I feel guilty today for not having been available to my family<br>Ongoing symptoms interfere with my life

Worry	Having had cancer makes me feel uncertain about my health<br>I worry about the future<br>Having had cancer makes me feel unsure about the future<br>I worry about cancer coming back<br>New symptoms make me worry about cancer coming back<br>I worry about my health<br>I feel like time in my life is running out

*Note: subscale range 1-5 (strongly disagree – strongly agree).
