# Supplementary material for: Cancer-related psychosocial factors and self-reported changes in lifestyle among gynecological cancer survivors: cross-sectional analysis of PROFILES registry data
Source: Support Care Cancer. 2021 Aug 28;30(2):1199–207. doi: 10.1007/s00520-021-06433-0 (PMC8727401; doi:10.1007/s00520-021-06433-0)
Supplement: Supplementary file 3 — Supplementary file3 (PDF 90 KB) [file 520_2021_6433_MOESM3_ESM.pdf]

Online Resource 3 Cancer-related psychosocial factors and self-reported changes in lifestyle among gynecological cancer survivors: cross sectional analysis of PROFILES registry data. *Supportive Care in Cancer*. Karin A. J. Driessen, MSc., Belle H. de Rooij, PhD, M. Caroline Vos, MD, PhD, Dorry Boll, MD, PhD, Johanna M.A. Pijnenborg, MD, PhD, Meeke Hoedjes, PhD, Sandra Beijer, PhD, Nicole P.M. Ezendam, PhD. Corresponding author: Nicole P.M. Ezendam, The Netherlands Comprehensive Cancer Organisation, n.ezendam@iknl.nl.

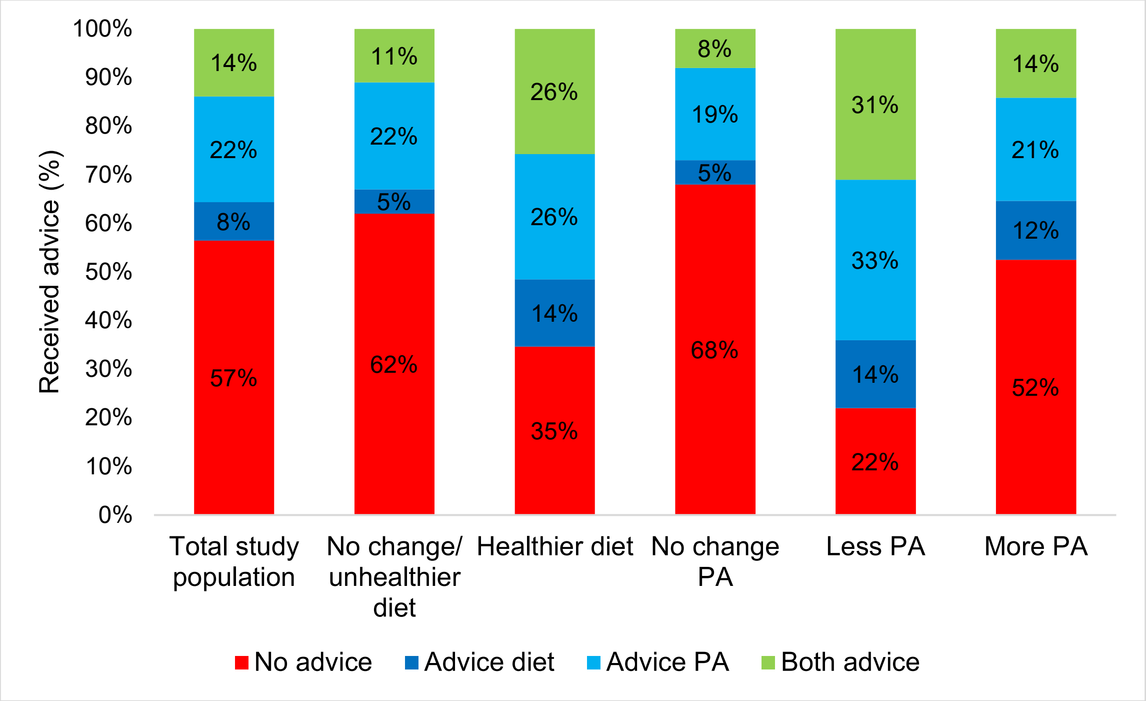

Fig. 1 Received advice regarding diet and/or physical activity in the total study population and according to change groups.

Note: percentages do not always add up to 100, since they were rounded up.
